# Correlates of intimate partner violence against women during a time of rapid social transition in Rwanda: analysis of the 2005 and 2010 demographic and health surveys

**DOI:** 10.1186/s12905-015-0257-3

**Published:** 2015-10-28

**Authors:** Dana R. Thomson, Assiatou B. Bah, Wilson G. Rubanzana, Leon Mutesa

**Affiliations:** School of Public Health, College of Medicine and Health Sciences, University of Rwanda, Kigali, Rwanda; Department of Global Health and Social Medicine, Harvard Medical School, Boston, MA USA; Isange One Stop Center Scale up Project, Rwanda National Police, Kigali, Rwanda; College of Medicine and Health Sciences, University of Rwanda, PO Box 945, Kigali, Rwanda

**Keywords:** Violence against women, Gender based violence, Domestic violence, IPV, GBV, DHS, Africa, Physical, Sexual

## Abstract

**Background:**

In Rwanda, women who self-reported in household surveys ever experiencing intimate partner violence (IPV) increased from 34 % in 2005 to 56 % in 2010. This coincided with a new constitution and majority-female elected parliament in 2003, and 2008 legislation protecting against gender-based violence. The increase in self-reported IPV may reflect improved social power for women, and/or disruptions to traditional gender roles that increased actual IPV.

**Methods:**

This is a cross-sectional study of IPV in 4338 couples interviewed in the 2005 and 2010 Rwanda Demographic and Health Surveys (RDHSs). Factors associated with physical or sexual IPV in the last 12 months were modeled using manual backward stepwise logistic regression. Analyses were conducted in Stata v13 adjusting for complex survey design.

**Results:**

Risk factors for IPV in 2005 (*p* < 0.05) were: experiencing emotional IPV (OR = 18.1), beating husband/partner unprovoked (OR = 12.3), witnessing IPV against mother (OR = 1.82), husband/partner consumes alcohol often (OR = 3.13), and polygynous marriage (OR = 1.51), whereas having a husband/partner with secondary education (OR = 0.43) was protective. Factors associated with increased IPV in 2010 (*p* < 0.05) were husband/partner (OR = 1.30) or woman (OR = 1.36) believes IPV is justified, husband/partner has sex with non-marital partners (OR = 2.52), bottom wealth quintile (OR = 1.25), polygynous marriage (OR = 2.29), having a son (OR = 2.05) or only daughters (OR = 2.58) versus no children, and having a husband/partner employed with in-kind versus cash compensation (OR = 1.58). In 2010, woman being involved with her own health (OR = 0.79) or earnings (OR = 0.57) decision-making was protective against IPV. Several variables were not available in the 2010 RDHS.

**Conclusions:**

Our results may provide evidence of both increased self-reporting of IPV and social power disruption. Rwanda’s Isange One Stop Center project, with medical, legal, and psychosocial services for domestic violence victims, is currently scaling to all 44 district hospitals, and police station gender desks reduce barriers to legal reporting of IPV. Additional support to *Abunzi* mediators to hear IPV cases in communities, and involvement of men in grassroots efforts to redefine masculinity in Rwanda are suggested. Additional research is needed to understand why self-reported IPV has increased in Rwanda, and to evaluate effectiveness of IPV interventions.

## Background

Intimate partner violence (IPV) describes physical, sexual, or psychological harm by a current or past partner. Not only does IPV compromise survivors’ basic human rights, physical and sexual assault can result in direct physical harm, sexually transmitted infections, or pregnancy, and all IPV can result in long-term mental and physical health problems [1, 2]. While IPV occurs in heterosexual and same-sex couples and is perpetrated by both women and men, the majority of cases are perpetrated by male partners against female partners worldwide [3]. A World Health Organization (WHO) analysis combining data from 77 studies across 56 countries estimated that, in Africa, 37 % of women have ever experienced physical or sexual IPV [3]. These rates were similar to the Eastern Mediterranean (37 %) and South-East Asia (38 %), and higher than the Americas (30 %), Europe (25 %), and Western Pacific (25 %) [3].

Within region and country, however, experiences of IPV vary widely, underscoring differences in national histories, institutional policies, cultural identities, resources, and other factors. When comparable measures and methods are used to measure lifetime prevalence of physical and sexual violence across national surveys [4–7], they show that lifetime prevalence of physical or sexual violence ranges from 64 % in Democratic Republic of Congo and Bolivia, to 6 % in Canada [3]. In national surveys, physical violence includes such actions as being beat, hit, kicked, choked, burned, or threatened with a weapon. Sexual violence is defined as being physically forced or threatened to have sex or to do something sexually degrading. Two such national surveys in Rwanda found that a woman’s experience of physical or sexual IPV in her lifetime almost doubled from 34 % in 2005 [8] to 56 % in 2010 [9] placing Rwanda among the countries with the high rates of IPV against women in the world.

Rwanda is a small, densely populated country that has undergone rapid demographic, social, and economic transition in the last 20 years since the Tutsi Genocide that killed around 1 million people. In the period between 2005 and 2010, fertility rates fell from 6.1 to 4.6 children per woman, child mortality was halved from 152 to 76 deaths per 1000 live births, and the percent of women completing secondary school increased from 1.6 to 4.3 % [8, 9]. Meanwhile, representation of women in parliament has increased dramatically from 18 % before the Genocide, to 26 % during the post-genocidal transitional government (1994–2003), to 56 % in the 2008 elections when Rwanda became the first and only country in the world with a majority woman parliament.

Women’s political representation and legal protection is an important step toward gender equity. Several factors may contribute to women’s political representation in Rwanda; foremost, Rwanda’s government has prioritized women’s political inclusion. In 2003, new legislation reserved 24 of 80 parliamentary seats for women-only parliamentarians to be filled by women-only voters [10]. While the evidence is mixed about whether more women representation changes policy outcomes overall [10], key pieces of legislation in Rwanda have certainly been shaped and shepherded by women parliamentarians including the 2008 law (No. 59/2008) on Prevention and Punishment of Gender-Based Violence (GBV). Some have suggested that Rwanda’s population, which is currently comprised of more women than men as a result of men being targeted during the genocide, has contributed to women’s political representation [10], as well as additional leadership roles by women within households [11]. Many assume that these gains in women’s representation and protections reflect women’s empowerment, which makes the dramatic rise in self-reported IPV against women between 2005 and 2010 particularly striking.

Violence in intimate partnerships is a common phenomenon worldwide, and is partially attributed to couples spending lots of time with each other [12]. Time exposure, however, does not explain why women and not men are most often the target of IPV. Feminist and socio-culture explanations provide frameworks to understand IPV against women. Power theory explains increases in IPV that coincide with women’s empowerment as a result of disruptions in traditional gendered roles [13]. Social learning theory adds that violence against women is learned through witness of IPV against women in childhood, and early experiences that cement these ‘lessons’ [14]. Background/situation modeling builds on this by adding that historical and socio-cultural context further normalizes violence in relationships [14].

In Africa, systematic gender inequality is often reinforced by cultural traditions of men in roles of head of household in charge of family finances and decisions [12], as well as colonial and post-colonial histories of slavery and labor migration that resulted in the absence of adult men and the feminization of poverty within households [13]. Until discussion of the new Inheritance Law began 1998, Rwandan land ownership and inheritance law treated women like minors; a spouse or father could appropriate a woman-owned business, and it was not acceptable for a woman to speak and share her own views in public (only on behalf of her family) [14]. The near universal exposure of adults to community violence during the genocide may exacerbate any existing ideas of normalized IPV. Finally, Rwanda faces the same male–female power differentials in the media as other countries [15]. Popular media worldwide portrays sexism, devaluation of women, and direct violence against women [16], which is reinforced locally by dominant community perceptions of gender differences [15, 17]. Adding household-level triggers for violence such as financial stress or alcohol abuse can further increase risk of IPV [14, 15].

Gender roles in Rwanda are in the midst of rapid transition, and the implications for IPV are not well understood. In a qualitative study in Rwanda after 2003, women described experiencing greater respect by family and community members, new confidence to speak in public forums, more autonomy and opportunities, as well as increased friction with their brothers and husbands, perceptions that men were withdrawing from politics, and feelings that the institution of marriage had been disrupted due to rapid changes in conventional gender roles [18]. A 2010 qualitative study on the same topic found that laws protecting women’s rights were perceived by women and men as having led to loss of women’s values and respect for men, thus provoking husbands to resort to violence to re-establish order in their households; this type of violence is believed by many woman and men as normal and even necessary [19, 20]. The large increase in self-reported IPV between 2005 and 2010 may reflect greater empowerment of women to speak about a high level of violence that already existed, or it could reflect a real increase in experiences of IPV. The present paper identifies factors associated with physical or sexual IPV in Rwanda in 2005 and 2010 that might inform hypotheses and further research about IPV against women in Rwanda.

## Methods

### Data

This analysis is based to the 2005 and 2010 Rwanda Demographic and Health Surveys (DHSs), which are nationally and sub-nationally representative two-stage cluster samples, conducted every 5 years to monitor demographic, socioeconomic, and health indicators [8]. In both surveys, primary sampling units (PSUs) were randomly selected from a recent census listing, and urban PSUs were oversampled to increase precision of urban estimates. Questionnaires were translated into Kinyarwanda, back translated into English, and field tested before implementation. Women aged 15 to 49 were the primary respondents, answering detailed questions about themselves, their households, and their children. Men aged 15 to 59 were secondary respondents, selected from every 3rd household in 2005, and every 2nd household in 2010. Both surveys were implemented by the National Institute of Statistics-Rwanda with technical support from Macro International, Inc. and funding from USAID, and in both surveys the all-female interviewing teams received the same standardized training [8, 9].

Of the 11,321 and 13,671 women interviewed in 2005 and 2010, respectively, 4066 and 5008 were randomly selected and agreed to complete a special module about domestic violence. Only one woman per household was selected for the domestic violence module to ensure that no one else in the household knew about sensitive questions that could compromise her safety, and to minimize the total number of women asked to describe traumatic events. Female interviewers received special training to conduct secure, confidential interviews in respondents’ homes and administered the domestic violence module in face-to-face interviews; they were supposed to skip the module if a confidential interviewing environment was not possible. According to the DHS datasets, 100 % of women in 2005 and more than 99 % of women in 2010 who were selected for the domestic violence module completed it. Of the 9074 women interviewed across the two surveys, 4338 had husbands/partners who were interviewed in the men’s survey. In this analysis of 4338 couples (2005: 1888; 2010: 2450), we link women’s self-reports of intimate partner violence in the last 12 months with husband/partner’s survey responses based on wife-ID in the men’s questionnaire.

In the domestic violence module, women were asked directly about their experiences of physical and sexual violence in the last 12 months and this outcome was modeled as a binary variable. In the 2005 survey only, women reported emotional IPV, physical violence perpetrated against her husband/partner when he was not already physically hurting her, history of her father beating her mother, and frequency of husband/partner’s alcohol usage, all of which are key risk factors for IPV against women [14, 21–25]. In both years, men and women were asked about their own demographic, education, and employment characteristics, as well as their perceptions of violence against women, and who makes decisions about their own health care and earnings. An adult in the household answered an additional questionnaire about household assets, and demographics of each household member including their age, sex, and household membership.

### Ethics

Both the 2005 and 2010 DHSs were reviewed and approved by the Macro International Internal Review Board, Rwanda’s National Institute of Statistics, and the National Ethics Committee of Rwanda. We were granted permission by Macro International, Inc. to use these de-identified data for this analysis.

### Data analysis

We used multivariable regression to identify belief or behavioral and socio-demographic factors associated with sexual or physical IPV against women in Rwanda in 2005 and 2010. We used percentages and Chi-square tests to compare (*p* < 0.05) socio-demographic characteristics of women in this study with married/partnered women not in the study (because their partner was not interviewed) and divorced or separated women. Then we defined 25 potential covariates and tested bivariate Chi-square distributions among women who had, and had not, experienced physical or sexual IPV in the last 12 months. Non-collinear (Pearson correlation *r* < 0.5) variables associated with IPV (at *p* < 0.1) were retained for multivariable model building. Finally, we used manual backward stepwise logistic regression, first removing variables that were least associated with IPV and retaining those variables that were associated with IPV (*p* < 0.05). Separate models were fit for 2005, for 2005 using the reduced set of variables available in 2010, and for 2010. The analysis was carried out in Stata version 13 using survey commands to apply sampling probability weights, account for clustering and stratification in the sample design, and perform subpopulation analysis in couples only. We presented final models as odds ratios (ORs) with 95 % confidence intervals.

## Results

Self-reported IPV in the last 12 months among married/partnered women doubled from 24.1 % to 49.5 % between 2005 and 2010. In 2005 and 2010, the married/partnered women in our study were slightly younger, less educated, and less likely to work than women whose husbands/partners were not interviewed or who were divorced or separated (*p* < 0.05 for all) (Table 1). There was a sizable increase in the percent of women who say a man is justified to beat his wife for at least one reason from 46.5 % in 2005 to 57.0 % in 2010 (Table 1).

**Table 1:** Distribution of key women socioeconomic characteristics and beliefs by marital status and inclusion criteria

	Interviewed about domestic violence					
	Included					
	% In union, partner interviewed	% In union, partner not interviewed	% Divorced, separated	X^2 ^ *p*-value	% Widowed	% Never in union
2005						
Woman’s age				<0.001		
15–19	1.1	0.1	0.7		0.0	60.0
20–29	39.9	30.9	30.5		3.0	33.7
30–39	35.4	38.1	32.5		27.4	4.6
40–49	23.5	30.9	36.4		69.5	1.6
Woman’s education				0.014		
No school	28.3	34.1	34.1		40.6	8.6
Primary	64.0	55.5	60.0		46.3	78.7
Secondary or higher	7.7	10.4	5.9		13.1	12.7
Woman’s employment				0.033		
Employed, for cash	17.0	18.0	24.7		24.9	19.6
Employed, in - kind	50.0	51.4	46.2		42.9	26.2
Not working	33.0	30.7	29.1		32.2	54.2
Household wealth				0.103		
Top 4 quintiles	79.6	76.2	74.5		71.2	80.9
Bottom quintile	20.4	23.8	25.5		28.8	19.1
Household residence				0.013		
Urban	12.0	14.6	17.6		19.9	21.6
Rural	88.0	85.4	82.4		80.1	78.4
Woman believes a man can beat his wife				0.822		
0 reasons	53.5	51.7	52.2		57.2	52.9
1+ reasons	46.5	48.3	47.8		42.8	47.1
Total	100.0	100.0	100.0		100.0	100.0
*N* (unweighted)	1888	448	378		157	1195
2010						
Woman’s age				<0.001		
15–19	1.4	1.4	3.0		0.0	54.5
20–29	42.5	30.0	30.4		5.2	39.7
30–39	36.0	38.5	35.8		21.0	4.5
40–49	20.1	30.1	30.8		73.8	1.3
Woman’s education				0.054		
No school	19.4	19.4	27.3		36.4	6.1
Primary	71.2	68.6	64.4		53.3	66.4
Secondary or higher	9.4	12.1	8.4		10.3	27.5
Woman’s employment				0.005		
Employed, for cash	56.9	52.8	65.9		60.5	34.6
Employed, in - kind	22.7	28.3	16.2		18.8	23.6
Not working	20.4	18.9	18.0		20.8	41.8
Household wealth				<0.001		
Top 4 quintiles	82.5	74.3	68.7		74.2	86.2
Bottom quintile	17.5	25.7	31.3		25.8	13.8
Household residence				0.697		
Urban	12.7	13.7	14.4		17.7	18.3
Rural	87.3	86.3	85.6		82.3	81.7
Woman believes a man can beat his wife				0.165		
0 reasons	43.0	43.8	36.7		43.4	47.7
1+ reasons	57.0	56.2	63.3		56.6	52.3
Total	100.0	100.0	100.0		100.0	100.0
*N* (unweighted)	2450	493	269		264	1532

In bivariate analysis in 2005, sexual or physical IPV was associated with emotional IPV (84.7 %), witnessing physical violence by father against mother as a child (31.5 %), and having a partner who consumes alcohol often (54.3 %) (*p* < 0.001 for all) (Table 2). IPV was also high in relationships where the wife reported beating her husband/partner when he was not already hurting her (82.4 %, *p* < 0.001), though only nine women (<1 %) reported this behavior. No other beliefs or behaviors were associated with IPV in 2005. A number of socio-demographic factors were associated with IPV in 2005 (*p* < 0.1) including rural residence, polygynous marriage, having any children, there being no adults other than the couple in the household, having a husband/partner with less than secondary education, and the woman having less than secondary education.

**Table 2:** Bivariate associations of physical or sexual IPV in the last 12 months with behavioral and socio-demographic characteristics

	2005				2010				Source Questionnaire
	*N* (weighted)	% IPV	95 % CI	X^2^	*N* (weighted)	% IPV	95 % CI	X^2^	
				*p*-value					
								*p*-value	
Beliefs and behaviors									
Emotional violence by partner last 12 months				<0.001					Women
No	1432	19.4	[17.4,21.5]		Not				
Yes	105	84.7	[76.4,90.5]		available				
Woman beat partner unprovoked last 12 months				<0.001					Women
No	1535	23.7	[21.6,25.9]		Not				
Yes	9	82.4	[49.0,95.8]		available				
Woman’s father beat mother				<0.001					Women
No	1028	20.4	[18.1,23.0]		Not				
Yes	507	31.5	[27.4,35.8]		available				
Partner consumes alcohol very often				<0.001					Women
Never or sometimes	1358	19.8	[17.8,21.9]		Not				
Very often	184	54.3	[46.5,61.8]		available				
Partner believes a man can beat his wife				0.188				0.003	Men
0 reasons	1196	23.2	[21.0,25.7]		1638	47.9	[45.2,50.5]		
1+ reasons	347	26.9	[22.2,32.3]		407	56.1	[51.5,60.6]		
Woman believes a man can beat his wife				0.108				<0.001	Women
0 reasons	828	22.5	[19.8,25.4]		879	43.7	[40.4,47.0]		
1+ reasons	718	25.9	[22.9,29.2]		1165	54.0	[50.9,57.0]		
Woman involved in her own health decisions				0.134				<0.001	Women
Partner only, other	654	26.1	[22.8,29.7]		553	56.6	[52.0,61.0]		
Woman involved	892	22.6	[19.9,25.6]		1494	46.9	[44.4,49.5]		
Woman involved with decisions about her earnings				0.249				<0.001	Women
Partner only, other	65	32.5	[23.5,43.0]		224	64.8	[58.0,71.1]		
Woman involved	217	26.1	[20.3,32.8]		1079	48.3	[45.2,51.3]		
No earnings	913	22.7	[20.0,25.6]		534	45.0	[40.7,49.4]		
Not working	347	24.8	[20.4,29.7]		206	51.4	[44.9,57.9]		
Partner had sex with non-wife last 12 months				0.552				<0.001	Men
No	1468	24.0	[21.8,26.2]		1971	48.7	[46.4,51.1]		
Yes	78	26.7	[18.5,36.8]		75	71.0	[60.9,79.3]		
Woman says it is okay to refuse sex				0.709				0.008	Women
No	485	24.7	[21.2,28.5]		445	55.4	[50.3,60.3]		
Yes	1059	23.8	[21.2,26.6]		1600	47.9	[45.4,50.4]		
Woman says it is okay to request use of condom				0.927				0.008	Women
No	848	24.0	[21.1,27.2]		445	55.4	[50.3,60.3]		
Yes	695	24.2	[21.1,27.6]		1600	47.9	[45.4,50.4]		
Socio-demographics									
Woman’s age				0.739				0.091	Women
15–19	18	18.7	[7.5,39.7]		29	29.9	[17.3,46.4]		
20–29	620	25.4	[22.3,28.8]		870	48.0	[45.0,51.0]		
30–39	547	23.4	[20.3,26.7]		737	51.3	[47.6,54.9]		
40–49	361	23.2	[18.5,28.8]		411	51.0	[45.1,57.0]		
Difference in partner and woman age				0.706				0.638	Women & Men
Woman older	238	25.4	[20.5,30.9]		360	50.2	[45.2,55.2]		
Same age, partner 0–4 years older	691	24.8	[21.7,28.3]		945	49.2	[46.0,52.5]		
Partner 5–9 years older	369	22.0	[18.1,26.4]		477	51.3	[46.6,55.9]		
Partner 10+ years older	248	23.9	[19.2,29.5]		265	46.5	[40.4,52.6]		
Household wealth				0.516				0.005	Household
Top 4 quintiles	1229	23.7	[21.5,26.1]		1689	48.2	[45.7,50.7]		
Bottom quintile	317	25.6	[20.7,31.1]		358	55.8	[50.8,60.6]		
Household residence				0.084				0.037	Household
Urban	187	19.4	[14.7,25.1]		259	43.4	[37.7,49.4]		
Rural	1360	24.7	[22.5,27.2]		1788	50.4	[47.9,52.9]		
Polygynous couple				<0.001				<0.001	Women
No	1388	22.3	[20.2,24.5]		1926	48.2	[45.9,50.5]		
Yes	156	40.2	[32.7,48.2]		116	70.0	[59.7,78.6]		
Woman’s children				0.040				<0.001	Women
Has no children	101	14.1	[8.7,22.1]		138	30.8	[23.1,39.7]		
Has at least one son	1195	24.9	[22.4,27.5]		1558	51.9	[49.2,54.6]		
Has daughters only	250	24.5	[20.0,29.6]		351	46.4	[41.6,51.2]		
Number of children <15 in household				0.229				0.003	Household
None	95	16.2	[10.4,24.4]		131	34.2	[27.0,42.2]		
1 or 2	592	23.7	[20.4,27.4]		881	49.7	[46.5,52.8]		
3 or 4	611	25.8	[22.5,29.5]		763	51.5	[47.9,55.2]		
5+	249	23.7	[18.6,29.7]		272	50.8	[44.3,57.2]		
Number of adults in addition to couple in household				0.087				0.167	Household
None	1042	26.1	[23.6,28.7]		1343	50.8	[48.3,53.3]		
1 or 2	393	20.8	[16.4,26.1]		558	48.8	[44.1,53.6]		
3+	111	16.8	[9.4,28.3]		146	40.2	[28.9,52.7]		
Partner’s employment				0.628				<0.001	Men
Employed, for cash	700	25.1	[22.0,28.4]		1630	47.7	[45.1,50.3]		
Employed, in - kind	202	23.9	[18.9,29.7]		390	58.4	[54.1,62.6]		
Not working	636	22.9	[19.8,26.4]		25	34.9	[17.4,57.7]		
Partner’s education				<0.001				0.020	Men
Less than secondary	1376	25.6	[23.3,28.0]		1804	50.6	[48.2,53.1]		
Secondary or higher	170	12.2	[8.3,17.7]		243	41.4	[34.3,48.8]		
Woman’s employment				0.204				0.002	Women
Employed, for cash	264	28.3	[23.2,34.1]		1165	52.9	[49.8,55.9]		
Employed, in - kind	773	23.7	[20.8,26.9]		464	45.1	[40.4,49.8]		
Not working	507	22.6	[19.2,26.4]		419	45.1	[40.8,49.5]		
Woman’s education				0.002				0.073	Women
No school	438	22.5	[18.9,26.6]		396	48.3	[43.1,53.5]		
Primary	988	26.1	[23.5,29.0]		1458	50.8	[48.1,53.5]		
Secondary or higher	121	13.2	[8.6,19.6]		193	42.1	[35.2,49.2]		
IPV overall^a^	1546	24.1	[22.0,26.3]		2047	49.5	[47.2,51.8]		

^**a**^Covariate frequencies may not add to total due to missing responses

In 2010, many of the same socio-demographic factors were associated with IPV, however, a number of beliefs and behaviors were newly associated with IPV (*p* < 0.1) including partner believes a man can beat his wife, woman believes a man can beat his wife, woman is not involved with her own health decision-making, woman is not involved with decision-making about her earnings, husband/partner has sex with non-wife partner(s), and the woman says she cannot refuse sex with her partner or request use of a condom.

In the 2005 multivariable analysis, two factors stand out as being strongly associated with IPV (Table 3). Women who experienced emotional IPV in the last year had 18 times the odds (*p* < 0.001) of sexual or physical IPV, and women who reported beating their husband/partner unprovoked had 12 times the odds (*p* < 0.01) of sexual or physical IPV. Having a partner that consumes alcohol very often (OR = 3.13, *p* < 0.001), witnessing physical IPV against her mother in childhood (OR = 1.82, *p* < 0.001), and being in a polygynous marriage (OR = 1.51, *p* < 0.05) were also associated with increased odds of sexual or physical IPV. Women who had a partner with secondary or higher education had lower odds of IPV (OR = 0.43, *p* < 0.01). When emotional violence, woman beats husband/partner unprovoked, woman witnessed IPV against mother in childhood, and husband/partner consumes alcohol often were removed from the model to make it comparable to the 2010 analysis, two additional demographic characteristics were associated with IPV: women’s primary education versus no education (OR = 1.40, *p* < 0.05), and woman has at least one son (OR = 2.04, *p* < 0.05) or daughters only (OR = 2.11, *p* < 0.05) versus no children.

**Table 3:** Multivariable odds ratios between risk factors and sexual or physical IPV in the last 12 months

	2005				2010	
	Full	Reduced	Full	Reduced	Full	Reduced
Beliefs and behaviors						
Emotional violence by partner last 12 months						
No	1.00	1.00			Not	Not
Yes	19.0***	18.1***			available	available
Woman beat partner unprovoked last 12 months						
No	1.00	1.00			Not	Not
Yes	10.8*	12.3**			available	available
Woman’s father beat mother						
No	1.00	1.00			Not	Not
Yes	1.80***	1.82***			available	available
Partner consumes alcohol very often						
Never or sometimes	1.00	1.00			Not	Not
Very often	3.16***	3.13***			available	available
Partner believes a man can beat his wife						
0 reasons	1.00		1.00		1.00	1.00
1+ reasons	1.11		1.12		1.29*	1.30*
Woman believes a man can beat his wife						
0 reasons	1.00		1.00		1.00	
1+ reasons	1.00		1.03		1.35**	1.36**
Woman involved in her own health decisions						
Partner only, other	1.00		1.00		1.00	1.00
Woman involved	0.80		0.84		0.78*	0.79*
Woman involved with decisions about her earnings						
Partner only, other	1.00		1.00		1.00	1.00
Woman involved	1.04		0.93		0.59**	0.57**
No earnings	0.71		0.64		0.48***	0.47***
Not working	0.75		0.72		0.65*	0.62*
Partner had sex with non-wife last 12 months						
No	1.00		1.00		1.00	1.00
Yes	0.94		1.13		2.49***	2.52***
Woman says it is okay to refuse sex						
No	1.00		1.00		1.00	
Yes	1.05		0.98		0.81	
Socio-demographics						
Woman’s age						
15–19	1.00		1.00		1.00	
20–29	1.15		1.22		1.47	
30–39	1.07		1.09		1.62	
40–49	1.48		1.33		1.89	
Household wealth						
Top 4 quintiles	1.00		1.00		1.00	1.00
Bottom quintile	0.98		0.99		1.22	1.25*
Household residence						
Urban	1.00		1.00		1.00	
Rural	1.44		1.17		1.06	
Polygynous couple						
No	1.00	1.00	1.00	1.00	1.00	1.00
Yes	1.55*	1.51*	2.35***	2.41***	2.31***	2.29**
Woman’s children						
Has no children	1.00		1.00	1.00	1.00	1.00
Has at least one son	1.95		2.04*	2.04*	1.67	2.05**
Has daughters only	2.24*		2.13*	2.11*	2.09*	2.58***
Number of children <15 in household						
None	1.00		1.00		1.00	
1 or 2	0.98		1.00		1.30	
3 or 4	0.99		1.20		1.23	
5+	1.04		1.17		1.18	
Number of adults in addition to couple in household						
None	1.00		1.00		1.00	
1 or 2	0.61*		0.67*		0.90	
3+	0.49		0.47		0.63	
Partner’s employment						
Employed, for cash	1.00		1.00		1.00	1.00
Employed, in - kind	0.77		0.76		1.54***	1.58***
Not working	0.72*		0.85		0.67	0.65
Partner’s education						
Less than secondary	1.00	1.00	1.00	1.00	1.00	
Secondary or higher	0.46**	0.43**	0.44**	0.45**	0.88	
Woman’s education						
No school	1.00		1.00	1.00	1.00	
Primary	1.34		1.38*	1.40*	1.30	
Secondary or higher	0.85		0.88	0.88	1.27	
*N* (weighted)^a^	1502	1520	1527	1544	2027	2029

***p < 0.001, **p < 0.01, *p < 0.5

^a^Sample size reduced by missing responses among covariates

Several beliefs and behaviors that were not associated with IPV in 2005 were significant in the 2010 multivariate analysis. Partner believes a man is justified to beat his wife (OR = 1.30, *p* < 0.05), woman believes a man is justified to beat his wife (OR = 1.36, *p* < 0.05), and partner has sex with non-wife partner(s) (OR = 2.52, *p* < 0.001) were all associated with greater odds of IPV. Furthermore, woman being involved with decisions about her own health (OR = 0.79, *p* < 0.05) or about her own earnings (OR = 0.57, *p* < 0.05) versus her partner alone were protective against IPV. Being a household in the bottom wealth quintile (OR = 1.25, *p* < 0.05), being in a polygynous marriage (OR = 2.29, *p* < 0.01), having a son (OR = 2.05, *p* < 0.01) or only daughters (OR = 2.58, *p* < 0.001) versus no children, and having a partner employed with in-kind versus cash earnings (OR = 1.58, *p* < 0.001) were associated with greater odds of IPV.

## Discussion

Physical or sexual intimate partner violence (IPV) self-reported in household surveys doubled between 2005 and 2010 which coincided with a rise in the percent of women who say that IPV is justified from 46.7 % to 57.0 %. In 2010, nearly half of all partnered women experienced physical or sexual IPV in the previous 12 months, and IPV tended to occur in tandem with multiple other forms of violence. Our 2005 finding that women who experienced emotional violence [26], who witnessed IPV as a child [21–24], or who were violent toward their husband/partner [26] were more likely to self-report IPV in the last year is consistent with other studies. The huge differential in experience of IPV among women versus male partners in 2005 suggests that violence against women is normalized. This is supported by surveys from around the world that find a high proportion of women and men believe that IPV against women is justified [17, 25]. We discuss two potential hypotheses for the sharp rise in self-reported IPV in Rwanda, and ways that individuals and communities might address IPV against women. Since women in this study were somewhat different from other partnered women, particularly divorced or separated women, caution should be used when generalizing these results.

### Individuals and couples

In our study, IPV was associated with high alcohol usage by husbands/partners, which is consistent with findings from diverse settings including Brazil, Kenya, and India [24, 27–29]. Other studies found that both woman’s and men’s alcohol usage was an important factor for IPV [22, 24]. Alcohol use directly affects cognitive and physical function, reducing self-control and leaving individuals less capable of negotiating a non-violent resolution to conflicts within relationships [30]. Excessive drinking by one partner can exacerbate financial difficulties, child abuse, infidelity or other stressful situations, which may fuel conflicts between partners. Because alcohol dependency is linked to numerous health and social problems for drinkers, their families, and communities [31], alcohol dependency is estimated to account for 4 % of global disability adjusted life years (DALYs) [32]. Responsible drinking campaigns, alcohol advertising bans, drinking and driving laws, and increased pricing of alcohol can be implemented by governments to deter drinking, and training of health, social, and legal professionals to support individuals who seek alcohol treatment can help to address IPV risk within couples [31].

Our 2010 finding that IPV was associated with husbands/partners who earned in-kind rather than cash compensation may provide evidence of social power disruptions within relationships as a source of increased incidence of IPV in Rwanda. In-kind rather than cash compensation may be related to the husband/partner having low education [21, 22, 33] which limits income potential, job security, and contributes to financial stress at home, a trigger for violence [28], whereas men who attend secondary school are typically exposed to ideas of human rights and gain skills for self-expression, which can reduce tolerance of IPV [28, 34]. In Rwanda, men have traditionally been heads of household, the primary cash earners, and decision-makers about household resources, however this has changed rapidly in recent years. Qualitative research about the impacts of Rwanda’s improved opportunities for women found that, in addition to numerous positive outcomes, non-submissive, independent women experienced increased conflict within their relationships when husbands felt their own roles were challenged or that their wives where skirting home responsibilities [18]. In India, South Africa, and elsewhere, increases in IPV have been linked with rapid changes in gender roles, including changes in husband/partner employment [31, 35]. In these settings, involvement of men in redefining masculinity has been important toward shifting public opinion about IPV and reducing incidence of IPV [34, 36]. The rise in reported acceptance of IPV among women in Rwanda suggests that women, too, are in need of involvement and support to redefine gender roles.

Certain family dynamics such as polygynous marriage and having children are slow to change in response to women’s changing roles in society, and it is therefore not surprising that these two factors remained significantly associated with IPV across years in our study. The practice of polygyny in Rwanda is illegal, uncommon, and may be falling [32]. In 2005, 12 % of married women were in a polygynous union, and in 2010, 8 % of women reported being in polygynous union [9]. Hypothesized links between polygyny and IPV are that the presence of more than two spouses contributes to more marital disagreement [37], and that the practice of polygyny reflects acceptance of male dominance in intimate partnership [38]. The link between having a child and increased risk of IPV among women could reflect that men who act violently toward their spouse/partner are also likely to act violently toward children [39]. Our finding that number of children in the household was not associated with IPV suggests that IPV in the context of having children is not instigated by economic or parenting stressors alone. Other studies found that in households with multiple forms of domestic violence including violence toward children, the level of violence against women sometimes increased if the woman directed aggression or neglect toward the child, or if she intervened in violence toward the children [40].

### Communities

While there is great variability in risk factors for IPV across countries, IPV beliefs and behaviors are commonly associated with IPV incidence [41, 42]. The addition of numerous belief and behavioral risk factors for IPV in the 2010 analysis may be evidence of improved reporting of IPV. Improved self-reporting during the time of this study is expected as a result of new laws and programs. Several laws passed between 1998 and 2008 addressed sexual violence used during the Genocide, the 2003 constitution created gender quotas and promoted gender equality, and the 2008 law on Prevention and Punishment of GBV made domestic violence illegal. To help overcome fears by women victims of IPV that their reports of violence will not be taken seriously, nearly all police stations staff a gender desk with trained, usually female, personnel. In 2009, the Rwanda National Police in partnership with the Ministry of Health and with the technical and financial support of UN agencies, namely UNICEF, UNWOMEN and UNFPA, launched the One Stop Center project that offers free, integrated medical, psycho-social, and legal services to victims of IPV and child abuse. In 2014, Isange One Stop Center Scale Up Project was launched with an aim of establishing this multidisciplinary service in all 44 district hospitals.

Given the complexity of IPV and the mixed evidence in this analysis, the higher incidence of IPV in 2010 may have resulted from both improved self-reporting and increased incidence of IPV due to social disruption. As such, we recommend multiple avenues to address IPV in Rwanda at a social-level. To the extent that prevalent violence against women already existed due to entrenched social, cultural, or historical norms, or has been inflamed by disrupting those norms, we recommend improved legal systems to protect women against violence, and campaigns to reduce acceptance of IPV and to redefine gender roles.

#### Extending legal protections

An alternative to the One Stop Center program is the *Abunzi* community mediation system [36] which was introduced in 2004 to deal with a backlog of cases in the court system, and has since been formalized with the creation of an *Abunzi* Secretariat which oversees mediator training and coordination with the Ministry of Justice. Like the *gacaca* courts, which heard genocide cases, the *Abunzi* system is a hybrid of traditional and modern methods of conflict resolution, and is perceived by many as more accessible and responsive than legal courts. Each *Abunzi* committee is comprised of at least 12 members, 30 % of whom are supposed to be women, and serve a local group of villages. While the *Abunzi* system may present fewer social barriers to victims of IPV than the justice system, many *Abunzi* committees do not maintain 30 % + women representation, mediations are public, and mediators are often unfamiliar with existing laws and turn to customary laws that may be prejudiced against women [41]. The *Abunzi* system may be a positive forum for certain IPV cases, especially if the victim is comfortable with the process, and the system is able to support couples to effectively communicate through differences that prevent future violence. However, better support and training of *Abunzi* mediators is needed to appropriately protect victims of domestic violence according to national law.

#### Redefining the roles of women and men in society

Pressure on men to change their behaviors and perception of themselves as men without well-formed alternative models of masculinity will not lead to sustained reductions in violence [28, 42]. This is likely true of women in terms of femininity, as well. As such, effective IPV policies and programs require men’s participation in redefining masculinity. Although much work has already taken place in Rwanda to redefine gender roles, it has happened relatively recently mostly through top-down approaches [14]. Successes include the 2008 law against gender-based violence which was brought to parliament by four women and four men who effectively fostered buy-in from the mostly male parliament at that time [43]. Other programs to prevent and respond to gender-based violence have been implemented within the Rwanda National Police and the Rwanda Defense Force, and gender quotas have been adopted for UN peace keeping forces [20]. Among the only grassroots initiatives on this issue in Rwanda, however, is the Men’s Resource Centre (RWAMREC) which is run by men who advocate for gender equity and promotion of non-violent ideals of masculinity [20].

Perhaps as a result of atypically top-down approaches, Rwandans overall hold highly inequitable attitudes about gender roles; for example, 61 % of Rwandan men agree with the statement “changing diapers, giving kids a bath and feeding kids are the mother’s responsibility” [44], and DHSs show increasing rather than decreasing tolerance for IPV. More bottom-up approaches may be needed in Rwanda. Grassroots approaches that have proven successful to shift attitudes about gender and IPV in other African contexts include single-sex group trainings with dialogues [45]. Although RWAMREC sensitizes communities to IPV laws during *umaganda*, the monthly national day of service, and facilitates dialogues about IPV in *umugoroba w’abashakanye* evening meetings of five to ten couples [20], further initiatives like these, along with time, may be needed to shift attitudes toward gender roles in Rwanda.

The doubling in self-reported IPV in Rwanda between 2005 and 2010 is deeply concerning, and further investigations of reasons for this increase are urgently needed. This study summarizes trends, correlates, and broad hypotheses for IPV, however, the cross-sectional nature of the DHS does not allow us to draw causal conclusions about predictors or consequences of IPV. Furthermore, measurement of physical and sexual IPV in household surveys is subject to under-reporting due to interviewer traits and difficulty securing privacy in smaller, densely populated homes [46]. We find it unrealistic that privacy was secured in 100 % (2005) and >99 % (2010) of interviews, and suspect that under-reporting of IPV occurred in both surveys. Finally, the 2010 DHS did not ask about emotional violence, IPV witnessed in childhood, woman’s violence against her husband/partner, and alcohol usage, all of which are important risk factors for IPV that could not be tested in our 2010 analysis.

The 2015 DHS was recently completed but not released at the time of this writing, and results from that survey might provide important insight about trends in IPV. Preliminary results of the 2015 DHS indicate continued rapid economic development in Rwanda, which is expected to correlate with a drop in IPV [12, 17]. We recommend additional research to tease out to what extent-increased self-reports of IPV are due to increased incidence of IPV versus greater empowerment by women to report violence. Researchers should investigate how changes in gender roles in Rwanda, and the unique demographic distribution of women and men in Rwanda, may relate to experiences of IPV. Furthermore, research is needed to evaluate the effectiveness of IPV interventions such as police station gender desks, district hospital Isange One Stop Centers toward reducing incidence of IPV. Concerns about how well *Abunzi* mediators are able to address IPV should be investigated. Campaigns that involve men and women in redefining gender roles could be an important contribution to the gender equality agenda moving forward in Rwanda, and the effects of these campaigns on IPV and IPV perceptions should be systematically evaluated.

## Conclusions

Rwanda has one of the highest self-reported rates of intimate partner violence against women worldwide, and multiple forms of current or past violence are reported by the same women. Doubling in self-reported violence between 2005 and 2010 coincided with major political and social gains. While the Rwandan health and legal sectors have multiple initiatives to support victims, additional campaigns may be needed to shift public perception and perpetration of intimate partner violence. The impact of these programs, and changes in reported violence, need further investigation.
